# De-implementation strategy to Reduce the Inappropriate use of urinary and intravenous CATheters: study protocol for the RICAT-study

**DOI:** 10.1186/s12879-016-2154-2

**Published:** 2017-01-10

**Authors:** Bart J. Laan, Ingrid J. B. Spijkerman, Mieke H. Godfried, Berend C. Pasmooij, Jolanda M. Maaskant, Marjon J. Borgert, Brent C. Opmeer, Margreet C. Vos, Suzanne E. Geerlings

**Affiliations:** 1 Department of Internal Medicine, Division of Infectious Diseases, Academic Medical Centre, Meibergdreef 9, 1105 AZ Amsterdam, The Netherlands; 2 Department of Medical Microbiology, Academic Medical Centre, Meibergdreef 9, 1105 AZ Amsterdam, The Netherlands; 3 Department of Internal Medicine, Academic Medical Centre, Meibergdreef 9, 1105 AZ Amsterdam, The Netherlands; 4 Department of Clinical Epidemiology, Biostatistics and Bioinformatics, Medical Faculty, Academic Medical Center, University of Amsterdam, Meibergdreef 9, 1105 AZ Amsterdam, The Netherlands; 5 Clinical Research Unit, Academic Medical Centre, Meibergdreef 9, 1105 AZ Amsterdam, The Netherlands; 6 Department of Medical Microbiology and Infectious Diseases, Erasmus University Medical Center, ‘s-Gravendijkwal 230, 3015 CE Rotterdam, The Netherlands

**Keywords:** Adult, Catheter-Related Infections/prevention & control, Urinary Tract Infections/prevention & control, Healthcare quality improvement, Implementation, Interrupted time series, Research Design

## Abstract

**Background:**

Urinary and (peripheral and central) intravenous catheters are widely used in hospitalized patients. However, up to 56% of the catheters do not have an appropriate indication and some serious complications with the use of these catheters can occur. The main objective of our quality improvement project is to reduce the use of catheters without an appropriate indication by 25–50%, and to evaluate the affecting factors of our de-implementation strategy.

**Methods:**

In a multicenter, prospective interrupted time series analysis, several interventions to avoid inappropriate use of catheters will be conducted in seven hospitals in the Netherlands. Firstly, we will define a list of appropriate indications for urinary and (peripheral and central) intravenous catheters, which will restrict the use of catheters and urge catheter removal when the indication is no longer appropriate. Secondly, after the baseline measurements, the intervention will take place, which consists of a kick-off meeting, including a competitive feedback report of the baseline measurements, and education of healthcare workers and patients. Additional strategies based on the baseline data and local conditions are optional. The primary endpoint is the percentage of catheters with an inappropriate indication on the day of data collection before and after the de-implementation strategy. Secondary endpoints are catheter-related infections or other complications, catheter re-insertion rate, length of hospital (and ICU) stay and mortality. In addition, the cost-effectiveness of the de-implementation strategy will be calculated.

**Discussion:**

This study aims to reduce the use of urinary and intravenous catheters with an inappropriate indication, and as a result reduce the catheter-related complications. If (cost-) effective it provides a tool for a nationwide approach to reduce catheter-related infections and other complications.

**Trial registration:**

Dutch trial registry: NTR6015. Registered 9 August 2016.

## Background

Healthcare-associated infections (HAIs) are associated with an increased mortality, a longer duration of hospital stay, which results into an increase in substantial costs. The use of invasive medical devices (e.g., urinary catheters, peripheral intravenous catheters (PIVCs) and central venous catheters (CVCs)) are important risk factors for the development of HAIs, which have prevalence of 7.1% measured in a combined point prevalence survey in Europe [1]. So an efficient way to reduce HAIs is to avoid insertion of catheters without an appropriate indication and to reduce the number of catheter days.

In general hospitals 15–25% of patients have an indwelling urinary catheter during their hospital stay. Urinary tract infections are accountable for 40% of all nosocomial infections in Western world hospitals, and 71–80% of these patients had a urinary catheter [2–4]. Nevertheless, the incidence of unwarranted placement of urinary catheters in hospitalized patients is 14–65% [5–10].

PIVCs are the most frequently used invasive medical devices in hospitalized patients. However, 25–56% of the PIVCs inserted in the Emergency Department are inappropriate or even unused [11–16]. In a recent study of internal medicine departments in Spain 81.9% of the patients had one or more PIVCs, of which 19% were no longer necessary [17]. A PIVC can cause serious adverse events, with an incidence rate of catheter-associated bloodstream infection of 0.1% (0.5 per 1000 catheter days) [18].

Central line-associated bloodstream infections (CLABSIs) are a major problem in intensive care units (ICUs). A meta-analysis shows that implementation of central line bundles to reduce the incidence of CLABSIs are effective and cost saving in ICUs [19].

### Intervention studies to prevent catheter-related infections

Previous research suggests that multiple and well-organized interventions could reduce the number of HAIs. In a pilot study in our university hospital in the Netherlands 89.2% of the initial indications for urinary catheter use were appropriate. However, after 2–3 days the initial indication was mostly no longer present, resulting into an inappropriate indication, but not to a removal of the catheter. After education and daily assessment of the indication of urinary catheters, the duration of catheterization reduced from 1009 to 672 days in 149 patients (pre-intervention *n* = 74, post-intervention *n* = 75), and the number of catheter-associated urinary tract infections (CAUTI) decreased from 4 to 0 infections per 1000 catheter days (*p* = 0.04). Thereby the median length of hospital stay reduced from 13 to 9 days [20]. Very recently, a national program (dissemination of information to sponsor organizations and hospitals, data collection, and guidance on key technical and socioadaptive factors) in 603 US hospitals reduced CAUTI rates by 22% in non-ICUs [21].

Only a few studies evaluated the effect of interventions to improve the appropriate use of PIVCs. In 1994 a quality improvement project in the internal medicine wards of Minnesota reduced inappropriate use of PIVCs by 63% (43% vs 27%) [22]. Education and feedback to improve PIVC care significantly reduced the PIVC-associated bloodstream infections from 2.2 to 0.44 per 10.000 patients days in 10 non-ICUs [23]. Furthermore, in a general hospital in Spain the use of unnecessary peripheral and central venous lines decreased from 22.9 to 7.1% after a 1-year training program [24].

A multifaceted ‘bundle’ approach (education, hospital protocol, national program, and checklist intervention) to control CVC-associated bloodstream infection in an internal medicine department in Spain showed a decrease of 63.1% (14.1 to 5.2 per 1000 catheter days) [25].

Prevention of HAIs is an important part of the todays medical practice. However, the risks of the use of urinary catheters and mainly PIVCs are widely underestimated, and in the Netherlands no nationwide program to reduce the catheter-related infections is present.

## Methods and Design

### Objectives

In this de-implementation study we aim for a 25–50% reduction of the number of urinary and (peripheral and central) intravenous catheters without an appropriate indication, which will lead to a reduction of the number of catheter days and catheter-related complications.

### Study design and setting

**Fig. 1:**
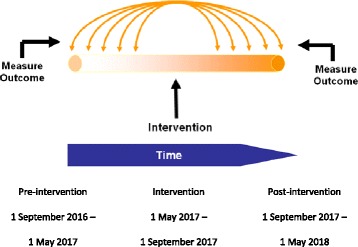
Interrupted time series for appropriate use of catheters

The study design is a multicenter, prospective interrupted time series (Fig. 1), which will take place in seven hospitals in the Netherlands (three university and four general hospitals). We aim to reduce 25–50% of the catheters without an appropriate indication by a de-implementation strategy of multiple interventions. The clinical data collection will be once per 14 days during 8 months in both the pre- and post-intervention period. During these measurement days all patients with a urinary and/or (peripheral and/or central) intravenous catheter inserted during the hospital stay will be enrolled. The de-implementation strategies will start during a transition period of 4 months, while no patients will be included.

### Appropriate use of catheters

We defined a list of valid indications for urinary and intravenous catheter placement, based on the literature (Table 1) [26–29]. All other indications are defined as inappropriate.

**Table 1:** List of appropriate indications

Urinary catheter	Peripheral intravenous catheter	Central intravenous catheter
Acute urinary retention or bladder outlet obstruction (≥150 cc)	Delivery of peripherally compatible infusate (IV fluids and medications), at least once in 24 h	Delivery of non-peripherally compatible infusate (e.g., irritants or vesicants), regardless of proposed duration of use
Accurate measurements of urinary output in critically ill patients required for treatment	Transfusion of blood and blood products	PICC : delivery of peripherally compatible infusate, with a duration of use which will likely confine ≥ 6 days^b^
Volume measurements of urine output aim for diagnostics (24 h urine), which cannot be assessed by other collection strategies	Injection of contrast fluids	Invasive hemodynamic monitoring or requirement to obtain central venous access in critically ill patients -Nontunneled CVC : duration of use will likely confine < 15 days -PICC : duration of use will likely confine ≥ 15 days
Assist in healing of open sacral or perineal wounds in patients with urinary incontinence	Intravenous access for cardiac dysrhythmia	PICC : Delivery of cyclical or episodic chemotherapy that can be administered through a peripheral vein, provided that the proposed duration of such treatment is ≥3 months
Continuous bladder irrigation for hematuria	PIVC : duration of use will likely confine ≤ 5 days	Frequent phlebotomy (every 8 h), provided that the proposed duration of such use is ≥6 days
Patient requires prolonged immobilization	Midline : duration of use will likely confine ≤ 14 days^a^	PICC : Intermittent infusions or infrequent phlebotomy in patients with poor/difficult peripheral venous access, provided that the proposed duration of such use is ≥ 6 days
Palliative care for terminally ill if needed		PICC: For infusions or palliative treatment during end-of-life care
Pre- or postoperative according (local) protocol		Tunneled catheter or port : preferred if proposed duration is ≥ 31 day

^a^Use of PIVCs is preferred over use of midlines for infusion of peripherally compatible infusates up to 5 days

^b^Use of PIVCs or midlines is preferred over use of PICCs for infusion of peripherally compatible infusates up to 14 days

### Definition of catheter-related infections

We will use the definitions by Centre for Disease Control’s (CDC) National Healthcare Safety Network for catheter-related infection (Table 2) [30]. Thereby we will use probable definitions based on the CDC and the Dutch surveillance system ‘Prevention of Nosocomial Infections through Surveillance’ (Table 3) [31].

**Table 2:** Definition of catheter-related infections [30]

Catheter-related infection	Definition
Healthcare-associated infection (HAI)	Infection where the date of event occurs on or after the 3rd calendar day of admission to an inpatient location, where calendar day 1 is the day of admission
Laboratory-confirmed bloodstream infection (LCBI)	Criterion 1: Patient has a recognized pathogen identified from one or more blood specimens by a culture or non-culture based microbiologic testing method which is performed for purposes of clinical diagnosis or treatment AND organism(s) identified in blood is not related to an infection at another site Criterion 2: Patient has at least one of the following signs or symptoms: fever >38.0 °C, chills, or hypotension AND organism(s) identified from blood is not related to an infection at another site AND the same common commensal (i.e., diphtheroids [Corynebacterium spp. not C. diphtheriae], Bacillus spp. [not B. anthracis], Propionibacterium spp., coagulase-negative staphylococci [including S. epidermidis], viridans group streptococci, Aerococcus spp., and Micrococcus spp.) is identified from two or more blood specimens drawn on separate occasions
Central line	An intravascular catheter that terminates at or close to the heart or in one of the great vessels (aorta, pulmonary artery, superior vena cava, inferior vena cava, brachiocephalic veins, internal jugular veins, subclavian veins, external iliac veins, common iliac veins, femoral veins) which is used for infusion, withdrawal of blood, or hemodynamic monitoring
Central line-associated BSI (CLABSI)	A LCBI where the central line was in place for >2 calendar days on the date of event, with day of device placement being Day 1, AND the central line was also in place on the date of event or the day before
Arterial or venous infection (VASC)	It must meet at least one of the following criteria: 1. Patient has organisms from extracted arteries or veins identified by a culture or non-culture based microbiologic testing method which is performed for purposes of clinical diagnosis or treatment. 2. Patient has evidence of arterial or venous infection on gross anatomic or histopathologic exam. 3. Patient has at least one of the following signs or symptoms: fever (>38.0 °C), pain, erythema, or heat at involved vascular site AND more than 15 colonies cultured from intravascular cannula tip using semiquantitative culture method. 4. Patient has purulent drainage at involved vascular site.
Urinary tract infection (UTI)	Patient has at least one of the following signs or symptoms: fever >38.0 °C, suprapubic tenderness, costovertebral angle pain or tenderness, urinary urgency, urinary frequency, dysuria^a^ AND urine culture with no more than two species of organisms identified, at least one of which is a bacterium of ≥10^5^ CFU/ml
Indwelling catheter (Foley catheter)	A drainage tube that is inserted into the urinary bladder through the urethra, is left in place, and is connected to a drainage bag.
Catheter-associated UTI (CAUTI)	A UTI where an indwelling urinary catheter was in place for >2 calendar days on the date of event, with day of device placement being Day 1, AND an indwelling urinary catheter was in place on the date of event or the day before

^a^An indwelling urinary catheter in place could cause patient complaints of frequency, urgency, or dysuria, and therefore these cannot be used as symptoms when catheter is in place

**Table 3:** Probable definition of catheter-related infections [31]

Catheter-related infection	Definition
Probable laboratory-confirmed bloodstream infection (LCBI)	Patient has at least one of the following signs or symptoms: fever >38.0 °C, chills, or hypotension AND organism(s) identified from (peripheral) blood or catheter segment is not related to an infection at another site AND defervescence within 48 h of catheter removal or initiation of appropriate antibiotic therapy
Probable central line-associated BSI (CLABSI)	A probable LCBI where the central line was in place for >2 calendar days on the date of event, with day of device placement being Day 1, AND the central line was also in place on the date of event or the day before
Phlebitis	Local pain, warmth, tenderness, erythema, and a palpable cord along the vein OR by positive sonographic examination in conjunction with erythema and edema of the extremity
Probable urinary tract infection (UTI)	Patient has at least two of the following signs or symptoms: fever >38.0 °C, suprapubic tenderness, costovertebral angle pain or tenderness, urinary urgency, urinary frequency, dysuria^a^ AND positive nitrite or leukocyte esterase dipstick test OR pyuria (>10 leukocytes/mm^3^) OR organism(s) seen in gram straining in not centrifuged urine OR two sequential urine culture (≥10^2^ CFU/ml) with the same uropathogens (gram negative bacteria or S. saprophyticus) OR urine culture with one species of organism identified (≤10^5^ CFU/ml) in a patient treated with appropriate antibiotic therapy OR the diagnose ‘urinary tract infection’ by doctor OR doctor starts appropriate antibiotic therapy
Probable catheter-associated UTI (CAUTI)	A probable UTI where an indwelling urinary catheter was in place on the date of event or the 7 days before

^a^An indwelling urinary catheter in place could cause patient complaints of frequency, urgency, or dysuria, and therefore these cannot be used as symptoms when catheter is in place

### Patient selection

**Fig. 2:**
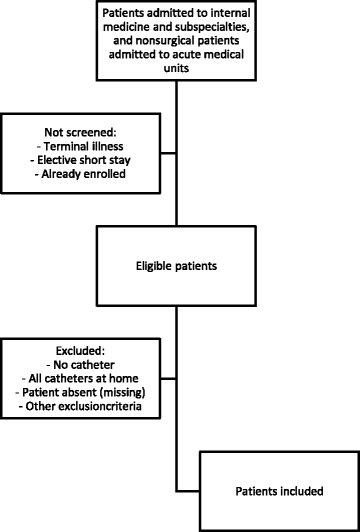
Flowchart of patient inclusion

We will include all patient (≥18 years old) admitted to internal medicine and subspecialties (gastroenterology & hepatology, geriatrics, pulmonology and rheumatology) and all nonsurgical patients admitted to acute medical units, who receive urinary and/or (peripheral and/or central) intravenous catheter. Patients admitted for elective short stay, terminally ill patients and patients who had all catheters prior to admission will be excluded (Fig. 2).

### Primary and secondary endpoints

The primary endpoint is the percentage of patients with an inappropriate indication for urinary and intravenous catheter on the days of data collection. Secondary endpoints are catheter-related infections and other complications, catheter reinsertion rate, use of antibiotics, length of hospital stay (and ICU) in days, in hospital mortality, and costs of the de-implementation strategy and the main healthcare costs.

### Assessments

The presence and indications for the catheter use will be extracted from the medical records in combination with observations of the admitted patients. During the day of measurement the indication of the urinary and/or intravenous catheter and some patient variables will be collected. If there is an unclear indication of a catheter, the investigator will contact one of the healthcare workers (HCWs) to verify this information.

After discharge, the occurrence of catheter-related infections (see Table 3 for definitions) [31], with diagnostics and treatment, or other catheter-related complications (measured in registry and by investigator), the number of catheter days, reinsertion rate, use of alternatives for an indwelling urinary catheter (continence garments, condom catheters, intermittent straight catheterization), reason for admission, Charlson comorbidity index [32], duration of hospital (and ICU) stay, readmission within 30 days after discharge, and mortality (in hospital or within 30 days after discharge) will be collected from (electronic) medical- and nursing records. Furthermore, the nurse-to-patient ratios and the clinical work experience of residents will be collected.

### De-implementation strategy

We will use a bundle of interventions (Table 4), since it is known that multiple and well-organized interventions result into better improvements. The main intervention is the dissemination of the list of appropriate indications (Table 1), which will restrict the insertion of urinary and intravenous catheters by physicians and nurses. The recommendation will be to remove the catheters without or with an expired valid indication. A local ‘champion’ (one of the leading physicians) will be appointed to be responsible for the interventions in his or her department. The intervention period will start with a kick-off meeting, where a list of baseline data of the intervention hospital in comparison with the other study hospitals will be presented as a competitive feedback report [33]. If desired, feedback reports per department could be sent by e-mail to local investigators. Thereby two educational meetings about insertion, care and maintenance of urinary and intravenous catheters will be applied to change HCWs behavior. Furthermore, HCWs will be encouraged and reminded to remove inappropriate catheters by posters, pocket cards and e-mail messages. In addition to other studies, patients will be actively supported by education material to participate in their treatment.

**Table 4:** De-implementation strategy

Interventions of de-implementation strategy
List of appropriate indications
Local ‘champion’
Kick-off meeting, including competitive feedback report of baseline data
Education meeting for healthcare workers
Education materials (e.g., posters, pocket cards)
Patient education materials
Optional interventions based on baseline data and local conditions per hospital

Subsequently, all impeded and promoted factors of this de-implementation strategy for both HCWs and patients will be evaluated by direct observations and interviews. Additional prevention strategies could be applied based on these affecting factors and local conditions in the different hospitals.

If this de-implementation strategy prove to be effective, persistent awareness of the local ‘champion’ in combination with recurrent surveillance of the appropriate use of catheters and catheter-related infections will improve the sustainability. Thereby the de-implementation rule “No indication = Remove catheter” will be recommended in national guidelines and local protocols.

### Sample size

The sample size is based on the objective of a 25–50% reduction in the number of invalid indications for both urinary and peripheral intravenous catheters, with a power of 80% and an alpha of 0.05. We excluded CVCs in the sample size, since the use of CVCs in the internal medicine and subspecialties departments is infrequent, but we will include all patients with a CVC in the study. We used the incidence of inappropriate use of the catheters from previous results in similar healthcare systems, which is 40% in urinary catheters [2, 20] and 50% in PIVC [13]. This results in 91–376 patients with a urinary catheter, and 66–284 patients with a PIVC. Correlating for 10–15% missing data the sample size is set to respectively 105–410 and 75–300 patients in both pre- and post-intervention group. This will result in a total sample size of 210–820 patients with a urinary catheter and 150–600 patients with a PIVC. We aim to include this number of patients in each hospital to evaluate the effect of the interventions in each individual hospital.

### Statistical analysis

Effect evaluation will be performed using SPSS statistics 23. Categorical variables will be presented as frequencies and percentages, and continuous variables as means (with a standard deviation) or medians (with an interquartile range) depending on the data distribution. We will use segmented regression analysis of interrupted time series methods to evaluate the differences between the baseline and intervention group [34, 35]. The data will be adjusted for possible confounders, autocorrelation and the underlying secular trend. We will perform stratified analyses to evaluate the impact of the de-implementation strategy in subpopulations. Figures will be used to visualize the underlying secular trend and the impact of the de-implementation strategy. The difference will also be presented in unadjusted and adjusted rate ratio (RR) with a 95% confidence interval (CI). Differences are considered to be statistically significant with a *p* < 0.05.

### Economic evaluation

The main question for the economic evaluation is if the benefits of a reduction in inappropriate use of catheters, which probably lead to a reduction in catheter-related infections, length of stay and associated costs, outweighed the costs associated with this de-implementation strategy. For feasibility reasons, we will use length of stay on general and ICU wards, readmission within 30 days of discharge, catheter-related complications, representing the main features of the healthcare system, and productivity costs for societal perspective. We will estimate the unit costs for healthcare service based on the prices in the Dutch guideline on healthcare costs [36]. We will divide the de-implementation costs in non-recurrent and recurrent costs. The non-recurrent costs are study-related, such as the costs of development of the de-implementation strategy, material costs, and costs of evaluation of the de-implementation. Recurrent costs are the costs to implement the de-implementation strategy. The primary analysis is a cost-effectiveness analysis, in which the difference in costs of the de-implementation and outcomes between the baseline and intervention group will be estimate using incremental cost-effectiveness ratios (ICERs). The cost-benefit analysis will be reported as the ratio of de-implementation costs to reduction of healthcare costs (de-implementation costs < reduction healthcare costs). Statistical uncertainty will be evaluated in sensitivity analyses, such as unit costs, preference weights, estimates of effectiveness and discount rate. The result from the economic evaluation will be extrapolated to the national level using a budget impact analysis according to the principles of the report of the International Society for Pharmacoeconomics and Outcome Research Task Force [37], conducted from societal perspective and health insurance or national health service perspective.

## Discussion

This study protocol describes the design, de-implementation strategy and evaluation of the ‘Reduce the inappropriate use of urinary and intravenous catheters’ (RICAT)-study. It could prevent the inappropriate use of urinary and intravenous catheters. If cost-effective it provides a tool for a nationwide approach to reduce catheter-related infections and healthcare costs.

A potential limitation of an interrupted time series design is the nonexistence of a control group. However, this quasi-experimental design is considered to be one of the most effective and powerful designs when randomization is not desirable or possible [34]. Another limitation is the inability to evaluate the impact of an individual intervention. In order to estimate the impact of a single de-implementation strategy there should be enough time between the different intervention periods, which is not possible in our study. Nevertheless, the interventions are well suitable for a broad de-implementation in other hospitals.

## Abbreviations

CAUTI: Catheter-associated urinary tract infection
CLABSI: Central line-associated bloodstream infection
CVC: Central venous catheter
HAI: Healthcare-associated infection
HCW: Healthcare worker
ICU: Intensive care unit
PIVC: Peripheral intravenous catheter
